# Reconstitution of Caruncle Placenta through the 20α-HSD/Casp-3 Apoptotic Pathway during Early Pregnancy in Bovines

**DOI:** 10.3390/cells12010162

**Published:** 2022-12-30

**Authors:** Ji-Hye Lee, Min-Gee Oh, Sang-Hwan Kim

**Affiliations:** 1 Institute of Applied Humanimal Science, Hankyong National University, Ansung-si 17579, Republic of Korea; 2 General Graduate School of Animal Life Convergence Science, Hankyong National University, Ansung-si 17579, Republic of Korea; 3 School of Animal Life Convergence Science, Hankyong National University, Ansung-si 17579, Republic of Korea

**Keywords:** apoptosis, progesterone, pregnancy, P4, ruminants, placenta

## Abstract

Trophoblast cells of endometrium during bovine pregnancy with different characteristics undergo dynamic changes during uterine remodeling, which can be observed as continuous changes, as P4 secreted by the mother is replaced by placental hormones. In this context, the present study analyzed tissues’ morphological changes through uterine apoptosis during early pregnancy. In addition, the expression pattern associated with apoptosis genes and 20α-HSD was determined in the endometrium and caruncle tissues. The localization of 20α-HSD, VEGF, Casp3, and mTOR protein was also determined in endometrium and caruncle during early pregnancy. From around 30 days, caruncle trophoblast cells with very high invasiveness expanded the villus section as the gestation period progressed. The surrounding cells detached and reorganized into new cells. In addition, an analysis of the effect of apoptosis on cell reorganization in the caruncle revealed that the expression of 20α-HSD/Casp-3 signals in the villus section gradually increased from 30 to 90 days. However, on the 30th day, glandular epithelial cells occurred sporadically in the trophoblast cell section. Moreover, the apoptosis of trophoblast cells increased at 90 days. Taken together, the results of the present study show that changes in the uterus during early pregnancy promote changes during later pregnancy by inducing the reorganization through the stimulation of 20α-HSD and Casp-3, promoting uterine and caruncle tissues, unlike cell development mediated by hormone signaling.

## 1. Introduction

During bovine pregnancy, the fusion of embryonic trophoblast and uterine endometrial cells is vital for fetal synchronization. In particular, the endometrial and caruncle epithelial cells, which undergo continuous changes during the first trimester, are important sites that maintain pregnancy and harbor signaling systems that regulate embryonic development. In ruminants, placental formation differs in function according to morphological changes, and the placental structure constitutes three different types of nutrient membrane cells (uninucleate trophoblast cells (UTCs, 80%), binucleate trophoblast giant cells (TGCs or BNCs, 20%), and hybrid fetomaternal syncytial plate cells (TNCs)) [1,2]. As pregnancy progresses, trophoblast cells differentiate into TGCs, which alter the characteristics of the placenta. In other words, the primary structure of ruminant placenta varies in function according to changes in epithelial cells that are directly attached to the fetal chorion. The uterine and placental epithelial cells of ruminants differ in terms of the action of hormones depending on the pregnancy period, thus helping control pregnancy and delivery through the induction of estrogen, progesterone, and prostaglandin synthesis via endocrine and autocrine mechanisms [3,4]. In addition, these hormones maintain the equilibrium between the fetus and uterus to maximize the release of angiogenic factors through rapid activation of the mTOR pathway, depending on the growth process of the developing fetus, or of transcription factors and homeobox proteins, which have been thought to affect development [5,6,7]. Specifically, the progesterone receptor (P4-r), which is activated in uterine epithelial cells for the functional division and differentiation of the uterus following implantation, may be involved in the regulation of uterine epithelial cell differentiation and substrate remodeling [8] through elevating the levels of intrauterine retinol-binding protein (RBP) [9,10]. Moreover, uterine remodeling undergoes continuous changes as P4 secreted from the gestational corpus luteum is replaced by placental hormones, and the prostaglandin factor 2 (PGF2) receptor in the uterus and placenta is activated during pregnancy [11,12]. Therefore, changes in the uterus affect the prostaglandin exchange between the mother and fetus through continuous changes [13]. In addition, changes in the uterus during early pregnancy are accelerated through alteration of the immune status of endometrial trophoblast cells [14,15]. For the success of this process, apoptosis must occur in the uterus and placenta at appropriate times. Hormone action and VEGF activity play essential roles in the process of tissue remodeling by apoptosis. In particular, the cell-cycle control protein (p27kip1), which is useful in forming endometriosis and rapid morphological changes in uterine tissue, controls apoptosis and VEGF activity [16]. In addition, the rapid activity of VEGF in the uterus promotes the proliferation of vascular epithelial cells around the tissue, forms new cytokines, and induces apoptosis in the surrounding tissue section to play a role in tissue reorganization [17,18]. However, reduced VEGF secretion in normal endometrial stromal cells (ESCs) of the stabilizing uterus appears to stabilize the tissue [18,19]. That is, the activity of VEGF is related to the apoptosis activity in tissue remodeling. However, on the contrary, low VEGF activity can be seen as playing a role in stabilizing tissues [16,18]. Kim et al. [7] observed 20α-HSD activity in the uterus and placenta during early pregnancy in cows, which dramatically altered the response to P4 hormonal stimulation; therefore, morphological changes during early pregnancy may be controlled by these factors [16,20]. Overall, changes in the uterus and placenta during early pregnancy appear to be closely linked to the maintenance of pregnancy. To this end, the present study analyzed morphological changes in tissues through apoptosis in the uterus and placenta during early pregnancy, as reported in existing studies of preimplantation tissue changes.

## 2. Materials and Methods

### 2.1. Preparation and Certification of Animals

The placentas and uteri of cattle were collected from a local slaughterhouse in Pyeongnong, Pyeongtaek, from each group of three Korean cattle at 30, 60, and 90 days of pregnancy, as previously reported. The harvested tissues were placed in cold phosphate-buffered saline (PBS; pH 7.2), transferred to the laboratory within 2 h, dissected into small pieces, and stored at −80 °C until use in the experiments. The present study was conducted in strict accordance with the recommendations of the Guide for the Care and Use of Laboratory Animals of the National Institutes of Health. All animal handling and experimental procedures followed a protocol approved by the Hankyong National University Animal Experimental Ethics Committee (IACUC approval HK−2018-1).

For protein and mRNA extraction, the fetal villi section of caruncle and the trophoblast cells/glandular epithelial cell section of endometrium were surgically excised and used.

### 2.2. Histological Examination of the Endometrial and Caruncle Tissues

All endometrial and caruncle tissues were collected and fixed in 70% diethyl pyrocarbonate (DEPC) ethanol, dehydrated, paraffin-embedded, and sectioned at 10 µm thickness. Representative sections of each endometrial and caruncle tissue paraffin block from all treatment groups were randomly selected and stained with hematoxylin and eosin (H&E; Sigma-Aldrich, St. Louis, MO, USA). The tissues were dehydrated, cleared, covered with Permount solution (Fisher Scientific, NJ, USA), and subjected to histological examination, using an optical microscope.

### 2.3. Immunofluorescence Detection

This experiment was conducted by applying the appropriate antibody dosage (10 μg/mL) and test method provided by Abcam int.

Paraffin blocks of uterine and caruncle tissues in each group were sectioned to a thickness of 5 μm. Tissue slides were prepared following deparaffinization, hydration using xylene and ethanol, and permeabilization at −20 °C with 0.1% Triton X-100 in 1 × PBS (PBS-T). The samples were blocked at room temperature (RT) for 30 min in TPBS (1 × PBS with 0.01% Tween-20) containing 5% normal horse serum (NHS) and 1% normal goat serum (NGS). All tissue slides were incubated in a dark room at RT for 1 h, with primary antibody 20α-HSD (Hankyong National University, Ansung, Republic of Korea) antibody diluted 1:200 in blocking solution. After that, to induce secondary antibody conjugation, the slides were incubated in the dark with a blocking solution for 30 min. After that, all slides were incubated with Alexa 594-conjugated anti-rabbit secondary antibody (ab150080; Abcam, Cambridge, UK) diluted 1:300 in blocking solution in the dark room at RT for 1 h. The second secondary antibody solution was then decanted and washed three times with PBS for 5 min (i.e., 5 min for each wash) in the dark. 

### 2.4. Immunofluorescence Method for Multicolor Staining of mTOR and VEGF Proteins

As described above, the first antibody, mTOR antibody (diluted 1:300 in blocking solution; sc-293133, Santa Cruz Biotechnology, Santa Cruz, CA, USA), was detected on the Caruncle tissue slide, blocked with blocking solution at room temperature in the dark for 30 min, and then conjugated with Alexa 488-conjugated anti-mouse secondary antibody (diluted 1:300 in blocking solution; ab150113; Abcam). For subsequent multicolor staining, second secondary antibody solution was decanted and washed three times with PBS, for 5 min each wash, in the dark. Then it was incubated for 1 h in the dark with blocking solution at room temperature again. Then the second primary antibody, VEGF antibody (diluted 1:300 in blocking solution; ab32152; Abcam), was detected in the dark room for 1 h on the Caruncle tissue slide. Then blocking solution was used in the dark at room temperature for 30 min, and Alexa 594-conjugated anti-rabbit secondary antibody was conjugated (diluted 1:300 in blocking solution) in the dark at room temperature for 1 h. The second secondary antibody solution was decanted and then washed three times with PBS, for 5 min each time, in the dark. All tissue slides after the experiment were incubated with cells on 0.1 μg/mL DAPI for 1 min. They were then rinsed with PBS. Afterward, the coverslip was mounted with a drop of mounting medium. The slides were mounted by using a fluorescent mounting medium (Dako, Carpinteria, CA, USA). Images of the detected protein were analyzed by using a fluorescence microscope (Olympus AX70).

### 2.5. Immunohistochemical Analysis of the Casp-3 Protein

For the immunohistochemical detection of Casp-3, tissues were prepared by deparaffinization and hydration, using the experimental methods mentioned above for 20α-HSD detection. Antigen retrieval was performed by heating the sections with 10 mM sodium citrate (pH 6.0) for 5 min at 95 °C. Endogenous peroxidase was quenched with 0.3% hydrogen peroxide in 1 × PBS for 5 min at RT. The slides were incubated in a blocking buffer containing 3% bovine serum albumin in 1 × PBS for 1 h at RT. The sample slides were incubated at RT for 2 h with antibodies against Casp-3 (ab4051, Abcam). The slides were cleaned with 1 × PBS and incubated at RT for 1 h with secondary anti-rabbit antibodies (sc-2054, Santa Cruz Biotechnology Inc., TX, USA). Following incubation, the sections were cleaned and activated for 10 min, using an ABC detection kit (Vector, CA, USA). Diaminobenzidine (Vector, CA, USA) was used as the substrate for horseradish peroxidase (HRP). The sections were stained with hematoxylin solution containing periodic acid–Schiff (PAS) reagent and 4% acetic acid. The tissues were dehydrated, cleared, and covered with Permount solution (Fisher Scientific, NJ, USA). The negative control of the immunohistochemical experiment was compared by detecting only the secondary antibody, excluding the primary antibody.

### 2.6. Non-Immune Controls of Immunolocalization Analysis

In all immunolocalization analyses, abnormal expression was compared by using a non-immune control group. In the non-immune control treatment, aberrant expression was confirmed by treatment with only the secondary antibody without treatment with the primary antibody [21].

### 2.7. In Situ End Labeling

For deparaffinization/hydration, the slides were emersed in xylene, 100% ethanol, and 95% ethanol two times for 10 min each; washed with double-distilled water for 5 min; treated with proteinase (10–20 μg/mL^−1^) for 15 min; and finally washed with 1 × PBS. After blocking with endogenous peroxidase (TaKaRa, Kusatsu, Japan) for 5 min (3% H2O2), the slides were washed with PBS. Next, 50 µL of labeling mixture (5 µL TdT enzyme + 45 µL Labeling Safe Buffer (TaKaRa)) was dropped onto the slides and allowed to react in a humid room at 37 °C for 60–90 min. Following incubation, the slides were washed with PBS three times, for 5 min each time, to stop the reaction. After reacting with 70 μL of HRP-conjugated anti-FITC (TaKaRa) at 37 °C for 30 min, the slides were washed with PBS three times, for 5 min each time, to stop the reaction. DAB was used to develop color at RT for 10–15 min, and the reaction was stopped with distilled water. After counter-staining with 3% methyl green, the slides were dehydrated, mounted, and observed under an optical microscope.

### 2.8. Alizarin Red Stain

To investigate the occurrence of possible calcium deposits within the endometrium and caruncle region, 5 μm sections of paraffin fixed tissues were incubated with Alizarin red stain for 3 min. The Alizarin red interaction with calcium results in an orange/red product that can be visualized under the microscope [22].

### 2.9. Real-Time PCR

Total RNA, extracted from the tissue scrapings by using TRIzol reagent (Cat No. 10296028, Invitrogen, CA, USA), was treated with DNAse (Ambion, Austin, TX, USA), as per the manufacturer’s instruction, and quantified by UV spectrophotometry. First-strand cDNA was synthesized by reverse transcription of mRNA, using Oligo (dT) primer and SuperScript II Reverse Transcriptase (Cat No. 18064022, Invitrogen). Real-time PCR was performed by using the One-Step SYBR Green RT-PCR Kit (Cat No. RR820A, TaKaRa), following the manufacturer’s protocol. Briefly, total RNA was isolated from tissues at each stage of bovine pregnancy, and 1 mg was used as the template. The primers used for PCR are listed in Table 1. The reaction conditions were as follows: 42 °C for 15 min; 95 °C for 2 min; 40 cycles at 95 °C for 40 s, 57–60 °C for 15 s, and 72 °C for 32 s; and final extension at 72 °C for 5 min. Rotor-Gene Real-Time Software 6.0 was used to analyze the cycle threshold (Ct) and to obtain a semi-log amplification plot. Finally, the relative expression levels of each gene were calculated by using the 2-ΔΔCt method by normalization with bovine glyceraldehyde-3-phosphate dehydrogenase (GAPDH) mRNA levels.

### 2.10. Efficiency of Primers of Real-Time PCR

Real-time PCR amplification efficiencies were generated by using pooled cDNA from all samples for each gene. Once you obtain their Ct values, plot them on a logarithmic scale, along with corresponding concentrations. PCR amplification efficiency was calculated by using the following equation: E = (10^−10/slope^−1) × 100 [23,24].

### 2.11. Western Blotting

For the Western blot, total protein was extracted from tissues, using Pro-prep solution (Intron, Seoul, Republic of Korea) according to the manufacturer’s instructions. Total protein was quantified by using Bradford protein assay (Bio-Rad, CA, USA), and the final protein samples were stored at −80 °C.

Protein extracts (30 µg) were separated in duplicate, using 13% sodium dodecyl sulfate–polyacrylamide gel electrophoresis (SDS–PAGE), and then transferred onto polyvinylidene fluoride (PVDF) membranes (Bio-Rad, Hercules, CA, USA). The membranes were blocked with 5% non-fat dry milk overnight at 4 °C and then washed for 10 min with TBS-T washing buffer (50 mM Tris–HCl (pH 7.6), 200 mM NaCl, and 0.1% (*v*/*v*) Tween 20). The membranes were incubated for 2 h with primary antibodies (1:1000 dilution), recognizing the active forms of 20α-HSD (Kim et al. [7]), Casp-3 (ab4051; Abcam, San Francisco, CA, USA), mTOR (sc-293133), and β-actin (sc-47778; all from Santa Cruz Biotechnology, Santa Cruz, CA, USA). Following primary antibody binding, the membranes were washed three times with TBS-T buffer for 15 min each and then incubated for 2 h with HRP-conjugated secondary anti-rabbit (sc-2054), anti-mouse (sc-2054 and sc-2031, all from Santa Cruz Biotechnology), or anti-goat (diluted 1:5000 in blocking buffer) antibodies. The membranes were incubated with the ECL detection reagent for 5 min in the dark and then exposed to X-rays for 10 min. Finally, relative protein expression was normalized to that of β-actin, which served as the internal control, using Alpha Innotech ver. 4.0.0 (San Leandro, CA, USA).

### 2.12. Statistical Analysis

Ratio analysis for protein expression in Western blotting experiments was quantified by using Excel (Microsoft Excel 2016). Real-time RT-PCR results were analyzed for statistical significance, using the SAS package (version 9.4; Statistical Analysis System Institute, Cary, NC, USA). Data were analyzed by using Welch’s *t*-test, fold change, and GLM in SAS. All data are presented as mean ± SEM. Differences between groups were considered significant at *p* < 0.05. All experiments were repeated three or more times.

## 3. Results

### 3.1. Morphological Changes in the Uterus and Caruncle during Early Pregnancy

**Figure 1:**
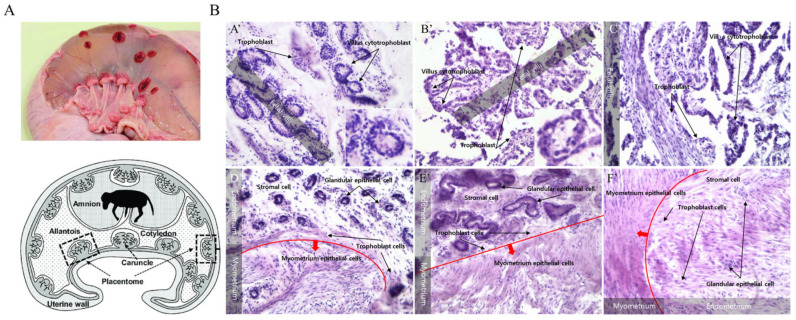
Histological analysis of bovine uterus and caruncle during early pregnancy. Microscope (Nikon Corp., Tokyo, Japan) magnification = ×200. (**A**) Anatomy of the caruncle [19]. (**B**) Histology.

The following changes were observed in the caruncle during early gestation at 30, 60, and 90 days. The gap between trophoblasts widened through fetal villi expansion, and the villus cytotrophoblast section expanded gradually. In particular, the fetal villus section expanded as the gestation period progressed, with very high connectivity between trophoblast cells at around 30 days. The surrounding cells detached and reorganized into new cells. In the case of the endometrium, the boundary between the stromal and glandular epithelial cell sections became clear at 30 days of early pregnancy, and the endometrium expanded until 60 days. Specifically, as the development of the glandular epithelial cell section progressed rapidly, it was left as a trace after 90 days and extended to the placentome. The development of the uterus and caruncle dynamically altered their morphology as pregnancy progressed. Therefore, the uterus and caruncle undergo tissue reorganization by expanding the trophoblast cell section between tissues (Figure 1).

### 3.2. Expression of Apoptosis-and Survival-Related Genes in Uterine and Caruncle Tissues

**Figure 2:**
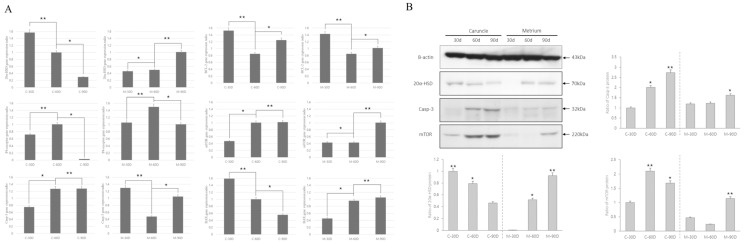
Expression analysis of apoptosis- and survival-associated genes in the endometrium and caruncle tissue in each group. (**A**) Real-time PCR analysis was repeated three times, and all data are expressed as mean ± standard error (*p* < 0.05). *, ** within the same column represent a significant difference. Gene expression data are normalized to bovine GAPDH (household gene) expression as the internal standard. (**B**) Western blotting data were quantified relative to β-actin (a housekeeping gene), and protein expression levels were analyzed by using Alpha Innotech, version 4.0.0.

In the analysis of apoptosis- and survival-related factors according to the gestation period, the expression of 20α-HSD and P4-r related to progesterone control differed between the uterus and caruncle. In the case of the uterus (Figure 2), 20α-HSD expression gradually decreased from days 30 to 90, whereas in the endometrium, its expression gradually increased until day 90 with morphological changes. In both the uterus and caruncle, P4-r expression was significantly increased at 60 days; however, it sharply decreased at 90 days in the caruncle, suggesting differences in hormonal effects. The apoptosis-related genes Casp-3, BAX, and BCL-2 were actively expressed in both the uterus and caruncle. However, BAX expression varied between the two tissues after 90 days of pregnancy (Figure 2A). The expression of mTOR, which acts as a cell survival signal, was increased in both the uterus and caruncle at 90 days. The results of the protein activity analysis were consistent with mRNA expression patterns. Specifically, after 30–90 days, 20α-HSD expression decreased in the caruncle but gradually increased in the uterus. Casp-3, a critical apoptosis-related factor, was highly expressed at 60 and 90 days in the caruncle. Conversely, in the uterus, its expression was slightly increased at 60 and 90 days compared with that at 30 days, albeit without significant difference. The protein expression of mTOR, a cell survival factor, gradually increased in the caruncle from 30 to 90 days. However, in the uterus, its expression peaked at 90 days (Figure 2B).

### 3.3. Detection of 20α-HSD Protein in Uterine and Caruncle Tissues

**Figure 3:**
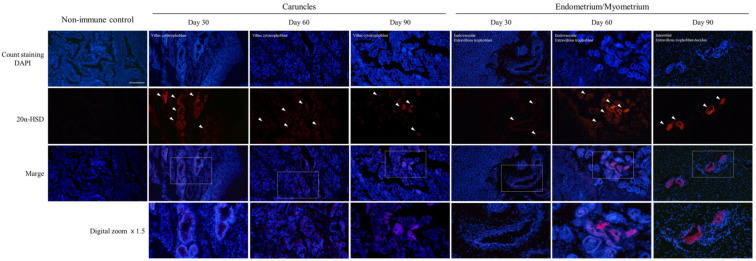
Immunofluorescence detection of 20α-HSD protein expression patterns in tissues during early pregnancy. The non-immune control was not treated with the first antibody; only the secondary antibody was treated to compare whether there was abnormal expression. White arrows indicate areas with distinct expressions. Red fluorescence indicates gene expression. Scale bar = 100 μm.

Expression of the 20α-HSD protein, which controls the action of P4, was analyzed by using immunofluorescence staining (Figure 3). At 30 days, 20α-HSD was detected in some cytotrophoblasts of the fetal villus section of the caruncle tissue. From 60 to 90 days, 20α-HSD detection in cytotrophoblasts in the connective tissue section in contact with the lumen increased gradually. In the endometrium, 20α-HSD was expressed in some glandular epithelial cells at 30 days, although the levels were trace compared with those at 60 and 90 days. At 60 days, 20α-HSD was highly detected in the glandular epithelial cell and connective tissue sections of the endometrium; in particular, 20α-HSD detection in the extravillous trophoblast decidua section gradually increased until 90 days. At 90 days, 20α-HSD was mainly detected in the interstitial extravillous trophoblast decidua. Overall, the detection pattern of 20α-HSD in uterine and caruncle tissues varied with the progression of the gestation period.

### 3.4. Expression of mTOR and VEGF Proteins in Caruncle Tissues

**Figure 4:**
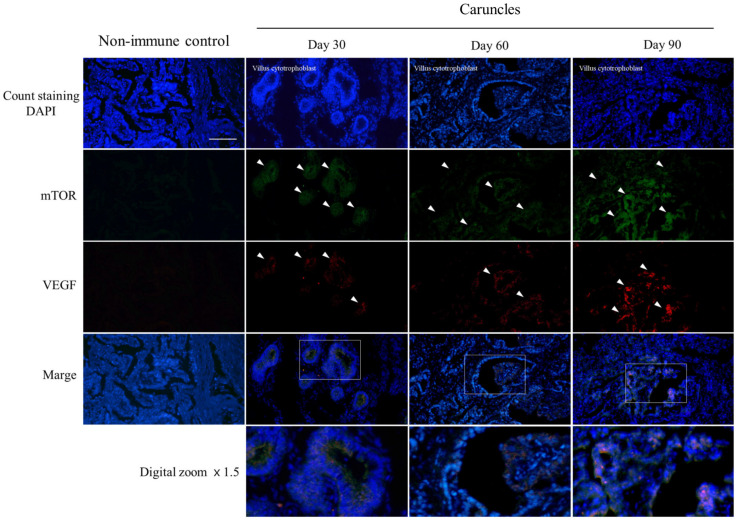
Immunofluorescence multicolor staining of mTOR and VEGF protein in caruncle tissue. Staining shows co-localization of detected protein with fetal villi and cytotrophoblast cells during early pregnancy caruncle tissue. Caruncle sections were stained with mTOR and VEGF antibodies in combination. mTOR was counterstained with Alexa Fluor 488 conjugated goat anti-mouse IgG (green). VEGF were counterstained with Alexa Fluor 594 conjugated goat anti-rabbit IgG (red). The nuclei were stained with Hoechst 33258 (blue). Overlays are shown in the down panels. Similar results were obtained in 3 separate experiments. White arrows indicate representative detection positions of the target protein. Scale bar = 100 μm.

As a result of the localization analysis of mTOR and VEGF protein detection in the caruncle, it was confirmed that mTOR and VEGF proteins were overall increased in the villus cytotrophoblast zone from 30 to 90 days (Figure 4). In the pattern of mTOR, expression increased in the overall area of the tissue centered on the villus cytotrophoblast in the fetal villi section at 30 days and 90 days, except for at 60 days. In contrast, the detection level increased remarkably at 90 days. This was similar to the detection pattern of VEGF, which is one of mTOR’s final protein translation functions. In particular, the detection of both proteins increased at 90 days compared to 30 and 60 days.

### 3.5. Apoptosis over the Pregnancy Period

**Figure 5:**
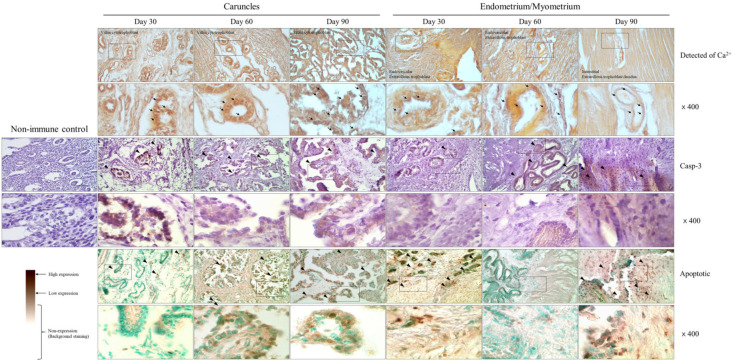
Expression and localization of calcium, Casp-3, and apoptotic label in the endometrium and caruncle tissue during early pregnancy. Endometrial tissue sections were immunostained with Casp-3 antibody and counterstained with hematoxylin. Brown indicates gene expression. In the apoptotic detection assay, terminal deoxynucleotidyl transferase was used to label the 3′-OH terminus of DNA fragments generated during apoptosis. For detection, HRP-conjugated anti-FITC (1:200) and DAB kit were used. Dark brown areas indicate the site of apoptosis. In the calcium analysis, the black arrows indicate calcium-positive trophoblast cells. Black arrows in other assays indicate the detection of proteins or apoptotic. Magnification = ×200, ×400. Scale bar = 100 μm.

As a result of analyzing the degree of accumulation of Ca^2+^ in the tissue, the concentration of both the endometrium and caruncle tissues from 30 days to 90 days seemed to increase and were centered on the trophoblast cells gradually, but other regions seemed to have a normal concentration. In particular, the concentration of Ca^2+^ in the caruncle rather than in the endometrium was observed to be higher in the villus cytotrophoblast and showed a distinct difference from other regions. In particular, it was increased more in the villus cytotrophoblast of the caruncle at 90 days of pregnancy and showed a low concentration in other sections (Figure 5). The analysis of apoptotic action for cell reconstitution in the caruncle confirmed that the detection of Casp-3, an apoptosis marker, continuously increased in the villus cytotrophoblast section from 30 to 90 days of pregnancy. In addition, our analysis of the apoptotic level revealed a similar pattern, although the degree of apoptosis was not high at 30 days, and it was mainly concentrated in the villus cytotrophoblast section at 60 and 90 days. The histological analysis revealed a similar pattern to that observed in the Western blotting. In the endometrium, Casp-3 was expressed in the glandular epithelial cell section at 30 and 60 days; however, in the myometrium zone, it was detected at 90 days. In the endometrium, apoptosis occurred sporadically in the tissue glandular epithelial and trophoblast cell sections at 30 days. Meanwhile, in the uterus, although the degree of apoptosis was very low in all sections at 60 days, its level increased in trophoblast cells at 90 days.

## 4. Discussion

In a previous study, 20α-HSD expression in the uterus was shown to vary depending on the gestation period, and this protein is implicated in the maintenance of normal pregnancy through the regulation of progesterone release and the switch from progesterone to placental hormone [7,14,25]. Therefore, changes in the uterus after implantation affect the functional ability of 20α-HSD. Specifically, these changes complete tissue reorganization, with active modulations of the homeobox mechanisms and apoptosis due to rapid changes in cell signaling in the endometrium during early pregnancy [26,27,28,29]. In common ruminants, the structure of the placenta is characterized by the fusion between the fetus and maternal epithelial cells after implantation, consisting of three completely different types of nutritional cells [2,30,31]. Among them, the continuous change of cytotrophoblast distributed in the fetal villus zone of the caruncle, which greatly affects fetal development, is very important for tissue reconstruction. In this study, a rapid morphological change of the caruncle was observed at 60 days, and cell changes were observed even at 90 days. In addition, it was confirmed that the cell activity increased as the concentration of Ca2 + increased in the fetal villus zone of the caruncle and the extravillous trophoblast of the endometrium. The extension of the uterine endometrial tissue section into the fetal villus zone during early pregnancy promotes the differentiation of cells present in the trophoblast zone, thereby successfully completing the maturity of the villus cytoplast section formed by the caruncle [32,33]. In particular, the glandular epithelial cell zone expands as the gestation period progresses, and the placentome undergoes morphological changes [25,27]. In addition, our findings demonstrated that changes in the caruncle affect the expansion of the placenta, as it is reorganized into a new tissue for the fetus through changes in the villus cytotrophoblast section during the first 30 to 90 days of pregnancy. During pregnancy, a phenomenon like this, the development of villus cytotrophoblasts and glandular epithelial cells, appears to increase the feedback action of progesterone through endocrine and autocrine mechanisms, thereby promoting pregnancy maintenance. However, 20α-HSD exerts an appropriate control action of the progesterone at 60 days of pregnancy to affect vascular expansion through the maternal junction, of the placenta and development of the placentome and consequent cell reorganization and is estimated to extensively form VEGF activity by mTOR [7,34,35]. Such dynamic tissue reorganization results in a selective apoptotic process during the functional modification of uterine cells, and this acts as a factor for reorganization throughout the tissue [36]. In this study, the action of 20α-HSD seems to be related to the activity of VEGF in tissue remodeling, and the relationship between VEGF and apoptosis at 90 days of pregnancy is related to new changes in the endometrium [17,19,35,36]. In addition, the action of 20α-HSD seems to act not only in the non-pregnant period but also in the pregnancy period, affecting changes in the endometrium and placenta [16,17,18]. In studies to date, many negative aspects related to tissue reorganization at the early stages of pregnancy according to apoptotic processes during morphological changes of the caruncle have been documented. However, for successful formation of caruncles in the uteri of ruminants during early pregnancy, differences in the development of the uterine body and caruncle affect the structural changes of TGCs, promoting tissue reorganization via the apoptotic mechanism [6,37]. This series of processes is thought to help maintain normal pregnancy by regulating progesterone signaling through an increase in 20α-HSD expression in the reconstituted tissue [27,38,39]. Unlike the hypothesis that cell apoptosis in the early stages of pregnancy will negatively affect pregnancy maintenance, this study estimates that 20α-HSD expression is induced to cause an appropriate level of cell apoptosis in order to actively cause tissue reconstruction in the early stages of pregnancy. In addition, it regulates apoptosis and the inhibitory action of progesterone differently in the endometrium and caruncle, thereby appropriately regulating tissue reorganization. In our study, tissue remodeling of the endometrium and caruncle during pregnancy did not occur in the same pattern. In other words, it was confirmed that the action of Casp-3 can be influenced according to the expression of 20α-HSD at different times. In addition, the action of 20α-HSD is considered to be regulated in a specific section rather than affecting the entire tissue. These results show that the expression pattern of Bax gene, which helps the activity of Casp-3, is the same as that of 20α-HSD, and the action of apoptosis factor in the tissue also shows a similar pattern. Our study showed that 20α-HSD and apoptosis act at entirely different times during the reconstitution process of the endometrium and caruncle, and they were highly active in the caruncle in the first 30 days of pregnancy, while they were highly active in the endometrium at 90 days. In other words, reorganization of the endometrium and caruncle after pregnancy occurs at different times, and it seems to induce the tissue reorganization of specific regions associated with the fetus. In particular, at 30 and 90 days of pregnancy, the expression of genes related to the programmed cell death and cell survival of cytotrophoblast in the tissues increases at a similar time, so it is thought that the two actions form a close relationship and regulate tissue reorganization. Compared to the study of Kim et al. [7], these results showed similar expression patterns of 20α-HSD in the ovaries and endometrium during the initial pregnancy period, and the caruncle seems to be affected from the 60th day of pregnancy. These results suggest that, at least in part, tissue reorganization begins in the endometrium, which is affected by the ovaries first, followed by caruncle reorganization. According to Ott [28], active immunological action occurs in the uterus and placenta during early pregnancy, and this may be involved in the maintenance of pregnancy through the activation and suppression of hormones (cytokines). This immune action may further cause appropriate apoptosis in the tissues [28,39,40]. In addition, structural changes in the caruncle through apoptosis regulate progesterone release into the placentome, induce functional changes in cells, and increase the activity of homeobox family proteins, all of which may affect cellular communication between the fetus and uterus [29,41]. From our study alone, there is no definitive result that 20α-HSD induces apoptosis in early pregnancy and thereby induces a cell-growth-related gene. However, it is clear that 20α-HSD can affect the reconstitution of the uterus during early pregnancy and is highly correlated with apoptosis. In conclusion, the present study showed that, in cows, morphological changes in the uterus and caruncle during early pregnancy depend on hormonal control and apoptosis. Our study showed that apoptosis in the uterus and caruncle during early pregnancy may play pivotal roles in the histological reconstruction and maintenance of pregnancy. Therefore, contrary to previous reports, we confirmed that the apoptotic mechanism during early pregnancy may in fact be involved in the smooth communication between the embryo and uterus.

## 5. Conclusions

The present study determined whether the different types of apoptotic activities of the uterus and caruncle from 30 to 90 days of gestation in cows affect tissue reorganization through specific hormonal control and effects on normal uterine morphogenesis. We confirmed that the expansion of the villus cytotrophoblast section in the primary cells of the uterus and caruncle is accelerated during early pregnancy, and dynamic tissue reorganization occurs in the surrounding structures. In particular, expansion of the intrauterine trophoblast section appears to produce a lasting effect on glandular epithelial cell activity. Moreover, 20α-HSD, activated around 60 days of pregnancy, likely promotes hormonal feedback and activates the progesterone synthesized in the placenta. Therefore, tissue reorganization through the apoptosis of uterine trophoblasts during early pregnancy promotes the function of the fetal villi of the caruncle to maintain a normal pregnancy.

## Figures and Tables

**Table 1:** Primers used for real-time PCR analysis of apoptosis-associated genes.

Primer Name	Sequence	Gene ID	Annealing Temperature (°C)	Amplification Efficiency (%)	Product Size (bp)
Bos 20α-HSD FW	5′- GCC ATT GCC AAA AAG CAC AAG -3′	NM_001167660.1	60	96	234
Bos 20α-HSD RV	5′- GGA AAG CGG ATA GTC AGG GTG ATC -3′				
Bos Casp-3 FW	5′-AGC CAT GGT GAA GAA GGA ATC A-3′	NM_001077840.1	60	97	137
Bos Casp-3 RV	5′- CCT CGG CAG GCC TGA ATA AT-3′				
Bos mTOR FW	5′-TCT CAT GGG TTT TGG AAC GA-3′	XM_005216989.1	60	93	111
Bos mTOR RV	5′-TGA GAG CTG TAC CCC AGC AG-3′				
Bos BCL-2 FW	5′-GAGTTCGGAGGGGTCATGTG-3′	NM_001166486.1	60	94	158
Bos BCL-2 RV	5′-GGGCCATACAGCTCCACAAA-3′				
Bos Bax FW	5′-GCCCTTTTGCTTCAGGGTTT-3′	NM_173894.1	57	96	179
Bos Bax RV	5′-ACAGCTGCGATCATCCTCTG-3′				
Bos P4-r FW	5′-TGG TTT GAG GCA AAA AGG AG-3′	NM_001205356.1	58	94	131
Bos P4-r RV	5′-CCC GGG ACT GGA TAA ATG T-3′				
Bos GAPDH FW	5′-GAAGGTCGGAGTGAACGGAT-3′	NM_001034034.2	60	98	180
Bos GAPDH RV	5′-TTCTCTGCCTTGACTGTGCC-3′				

## Data Availability

Not applicable.
